# Unc45b Forms a Cytosolic Complex with Hsp90 and Targets the Unfolded Myosin Motor Domain

**DOI:** 10.1371/journal.pone.0002137

**Published:** 2008-05-14

**Authors:** Rajani Srikakulam, Li Liu, Donald A. Winkelmann

**Affiliations:** Department of Pathology and Laboratory Medicine, Robert Wood Johnson Medical School, University of Medicine and Dentistry of New Jersey, Piscataway, New Jersey, United States of America; Swiss Federal Institute of Technology Lausanne, Switzerland

## Abstract

Myosin folding and assembly in striated muscle is mediated by the general chaperones Hsc70 and Hsp90 and a myosin specific co-chaperone, UNC45. Two UNC45 genes are found in vertebrates, including a striated muscle specific form, Unc45b. We have investigated the role of Unc45b in myosin folding. Epitope tagged murine Unc45b (Unc45b^Flag^) was expressed in muscle and non-muscle cells and bacteria, isolated and characterized. The protein is a soluble monomer in solution with a compact folded rod-shaped structure of ∼19 nm length by electron microscopy. When over-expressed in striated muscle cells, Unc45b^Flag^ fractionates as a cytosolic protein and isolates as a stable complex with Hsp90. Purified Unc45b^Flag^ re-binds Hsp90 and forms a stable complex in solution. The endogenous Unc45b in muscle cell lysates is also found associated with Hsp90. The Unc45b^Flag^/Hsp90 complex binds the partially folded myosin motor domain when incubated with myosin subfragments synthesized in a reticulocyte lysate. This binding is independent of the myosin rod or light chains. Unc45b^Flag^ does not bind native myosin subfragments consistent with a chaperone function. More importantly, Unc45b^Flag^ enhances myosin motor domain folding during *de novo* motor domain synthesis indicating that it has a direct role in myosin maturation. Thus, mammalian Unc45b is a cytosolic protein that forms a stable complex with Hsp90, selectively binds the unfolded conformation of the myosin motor domain, and promotes motor domain folding.

## Introduction

Folding of myosin in striated muscle follows a pathway mediated by the molecular chaperones Hsp90, and Hsc70. Newly synthesized myosin forms a transient complex with these general chaperones in which the myosin motor domain is partially folded [1]. Myosin transits through this chaperone complex on the pathway to myofibril assembly. This pathway appears to involve an Hsp90 co-chaperone, Unc45, found in both invertebrates and vertebrates. There is a single invertebrate *unc-45* gene in *C. elegans* and *Drosophila* [2]. Temperature sensitive alleles of the gene disrupt thick filament assembly in the body wall muscles [3]–[5]. Maternal Unc-45 in early embryos is involved in cytokinesis and co-localizes with non-muscle myosin [6]. Vertebrates express two distinct paralogs of *C. elegans unc-45* [7]. One, *unc45a* (formerly GC-UNC45), is expressed generally in all tissues, and expression of the other, *unc45b* (formerly SM-UNC45), is limited to striated muscle. In zebrafish embryos, depletion of Unc45b results in paralysis and cardiac dysfunction in embryos that is correlated with a loss of myosin filaments in sarcomeres. These results are consistent with a role in assembly of muscle specific myosin isoforms required for cranial, cardiac and skeletal muscle contraction [8].

The *unc45b* gene encodes a ∼103 kDa protein (Unc45b). The protein has three basic motifs: an amino terminal region with three tetratricopeptide repeats (TPR), a central region of unknown function, and an approximately 420 residue carboxyl terminal region called the UCS domain that is shared by proteins that interact with myosin. The TPR motif is a protein-protein interaction module of 34 amino acids that is often found in tandem repeats of 3–16 units [9]. The UCS domain is named for the three founding protein family members, UNC-45 from *C. elegans*, CRO1 from the filamentous fungus *Podospora anserina* [10] and She4p from *S. cervisiae* [11]. Mutations in the UCS domain result in decreased accumulation and altered assembly of type II striated muscle myosin filaments [4], disruption of contractile ring formation [12], and disorganization of the actin cytoskeleton [10], all activities that are dependent on myosin function. The interaction of Hsp90 with UNC-45 via the TPR motif has been demonstrated *in vitro*, and the carboxyl-terminal regions of UNC-45 bind to and block thermal aggregation of the myosin head suggesting a role for UNC-45 as an Hsp90 co-chaperone with myosin binding activity [13].

We have expressed recombinant epitope tagged murine Unc45b in a mouse myogenic cell line and in bacteria and have isolated and characterized the protein. We show here that Unc45b is a cytosolic protein in eukaryotic cells and isolates as a stable complex with Hsp90. Unc45b^Flag^ expressed in bacteria is a soluble monomeric protein that readily forms a complex with purified Hsp90. Pure Unc45b^Flag^ is an elongated molecule when visualized by EM. The Unc45b/Hsp90 complex specifically binds the unfolded myosin motor domain but not native myosin, binding interactions that are characteristic of a chaperone complex. More importantly, we show that the Unc45b/Hsp90 complex dramatically enhances the folding of newly synthesized smooth muscle myosin motor domain placing this complex on the pathway leading to myosin maturation.

## Materials and Methods

### Materials

Anti-Flag M2 mAb agarose beads, 3× Flag elution peptide, bovine pancreas trypsin, and Protease Inhibitor cocktail were obtained from Sigma-Aldrich Chemicals (St. Louis, MO) and used as per manufacturer's directions. Purified human Hsp90 was from Assay Designs (Ann Arbor, MI), and rabbit polyclonal anti Hsp84 (Hsp90β) antibody was purchased from Lab Vision (Fremont, CA). All secondary antibodies used in Western blotting, as well as ECL chemiluminescence reagents were purchased from GE Healthcare (Piscataway, NJ). Coupled transcription and translations were performed with TNT Quick kits purchased from Promega (Madison, WI), and supplemented with Redivue L-[^35^S] Methionine (GE Healthcare).

### Vector Construction

The full length cDNA for Unc45b was cloned by RT-PCR from mature C2C12 myotube poly-A^+^ RNA. The sequence was confirmed by bidirectional sequencing and comparison to the mouse genome. The Unc45b cDNA was cloned 5′ to 3′ between the Not I and Xho I restriction sites of the pShuttle-IRES-hrGFP-1 vector (Stratagene, La Jolla, CA) placing the full length cDNA with its native translation start site under control of the shuttle vector CMV promoter. The 3′ end of the cDNA is fused in frame with a 3× Flag epitope coding sequence with a two amino acid linking sequence (leu-glu). This is followed by an IRES sequence that directs internal ribosome initiation of a sea pansy GFP coding sequence and is terminated with a SV40 Poly A signal sequence. The shuttle vector was used to prepare replication deficient adenovirus using the AdEasy system (Stratagene) as previously described [14], [15]. The Unc45b^Flag^ coding region was cloned from an Nco I restriction site at the initiation codon of Unc45b^Flag^ through a Not I site introduced after the Flag tag termination codon. The insert was cloned into the Nco I – Not I sites of pET-21d (Novagen, Darmstadt, Germany).

All of the myosin expression vectors used in coupled translation assays contain the coding sequences downstream of an SP6 promoter in a pGEM4 vector. Coupled transcription and translation assays were incubated 2 hr at 30°C with 2 µg plasmid DNA per 50 µl reaction. Construction of the vectors for the embryonic chicken skeletal muscle full length myosin heavy chain (MHC), heavy meromyosin subfragment (HMM, 1-1293), myosin Subfragment 1 (S1, 1-845), subfragment 2 (S2, 846-1293), MD::GFP chimera (Sk_795_GFP), and the essential and regulatory myosin light chains have been described in detail elsewhere [14], [16]. The cDNA encoding chicken gizzard smooth muscle myosin motor domain was provided by Kathy Trybus, University of Vermont [17]. The design of smooth muscle MD::GFP chimera (Sm_795_GFP) is identical to the Sk_795_GFP vector with the junction between the MD and GFP at conserved sequences within the light chain binding helix. The smooth muscle MD::GFP chimera vector was confirmed by bidirectional sequencing and produced a 116 kDa protein when expressed in the coupled translation assay.

### Muscle cell expression and purification of Unc45b^Flag^

Maintenance of the mouse myogenic cell line, C2C12 (CRL 1772; American Type Culture Collection, Rockville, MD), has been described in detail elsewhere [14], [18]. Sub-confluent C2C12 myoblasts are infected with replication defective recombinant adenovirus (Ad-Unc45b^Flag^/GFP) at 5×10^8^ pfu/ml in growth medium (90% DMEM, 10% FBS) by incubation of 2 ml of virus per p100 dish of cells for 2 hr at 37°. Each dish is supplemented with 6 ml of medium and incubation with virus is continued overnight. Twenty hours post-infection, cells are transferred to fusion medium (89% DMEM, 10% horse serum, 1% FBS) to induce differentiation. Expression of recombinant Unc45b^Flag^ is monitored by accumulation of GFP fluorescence in infected cells. Myocyte differentiation and fluorescence accumulation are monitored for the next 96–120 hrs until the cells are harvested. Cells are chilled, media is removed, and the cell layer is rinsed three times with cold PBS. The cells are scraped into 0.5 ml/dish of triton extraction buffer (100 mM NaCl, 0.5% Triton X-100, 10 mM Imidazole pH 7.0, 1 mM DTT, 5 mM MgATP, and Protease Inhibitor cocktail). The cell suspension is collected in an ice-cold Dounce homogenizer and lysed with 15 strokes of the A pestle. The cell debris in the whole cell lysate (WCL) is pelleted by centrifugation at 17,000×g for 15 min at 4°C. The triton soluble extract (TSE) is fractionated by ammonium sulfate precipitation using 0–30% saturation, and 30–60% saturation. Unc45b and Unc45b^Flag^ precipitate between 30–60% saturation of ammonium sulfate. The recovered pellet is dissolved in 1/10 of the original the volume of buffer and dialyzed against 150 mM NaCl, 50 mM Tris-HCl pH 7.5 (TBS).

Anti-Flag M2 mAb coupled agarose beads are washed according to the manufacturer's recommendations, and dialyzed Unc45b^Flag^ containing fraction is incubated with 100 µl of a 1∶1 slurry of M2 agarose beads overnight at 4°C. Protein bound to anti-Flag agarose beads is collected by brief centrifugation at 1,000×g. Beads are washed with 1 ml of TBS for 40 minutes at 4°C for the first wash, followed by three washes with the same buffer for 10 min each. Bound protein is recovered by four to five successive elutions with one column volume each of 100 µg/ml 3× Flag peptide. For some pull-down assays Unc45b^Flag^ was not eluted and used bound to the agarose beads. Unc45b bound to beads is stored at 4°C suspended in an equal volume of TBS.

### Unc45b^Flag^ binding assays

100 µg of bacterial expressed and purified Unc45b^Flag^ (Fig. S1) is bound to 100 µl of M2 agarose in 500 µl of TBS as described above. Simultaneously 100 µl of M2 agarose is incubated with an equal volume of 100 µg/ml 3× Flag peptide. Aliquots (15 µl) of M2 beads/Unc45b^Flag^, or M2 beads/Flag peptide are incubated with 3 µg purified human Hsp90 in 25 µl TBS or 50 µl rabbit reticulocyte lysate for 45 min at 22°C. Samples were washed four times with 1 ml TBS and extracted into 40 µl of SDS-PAGE gel loading buffer. The supernatant and pull-down fractions were analyzed by SDS PAGE [19]. To avoid overloading, rabbit reticulocyte lysate was diluted 1∶20 for the gels. Unc45b^Flag^ binding to native HMM was assayed in the same manner except, aliqouts (15 µl) of M2/Unc45b^Flag^, or M2/Flag peptide are incubated with 2 µg purified chicken muscle HMM alone or with 3 µg of purified human Hsp90.

Binding of myosin subfragments to Unc45b^Flag^ and Hsp90 used radioactive subfragments synthesized in a 75 µl coupled translation assay containing 3 µg of plasmid DNA and incubated for 2 hours at 30°C. The reaction was split into 3 aliquots and incubated for 45 min at 22°C with 20 µl suspension of anti-Flag beads alone, or anti-Flag beads with bound Unc45b^Flag^ or with bound Unc45b/Hsp90 complex. The beads were pelleted by brief centrifugation and then washed four times with 1 ml TBS. Proteins bound to the beads were eluted into 20 µl of SDS gel loading buffer and analyzed by SDS-PAGE followed by autoradiography.

To investigate the concentration dependence of Unc45b binding to nascent myosin motor domain and Hsp90, the skeletal muscle MD::GFP was synthesized in the coupled assay for 2 hr at 30°C. Then the reaction was aliquoted and incubated with increasing concentrations of Unc45b^Flag^ (0–2000 nM) for 1 hr at 25°C. Anti-Flag beads (20 µl) were added and incubated for an additional hour at 25°C, washed and the bound proteins eluted into 20 µl SDS gel loading buffer and analyzed by SDS-PAGE and autoradiography.

### Gel filtration and immunoprecipitation of endogenous Unc45b

A 30–60% saturation ammonium sulfate fraction was prepared from fully differentiated C2C12 myotubes as describe earlier and dialyzed against 50 mM Tris-HCl pH 7.4, 150 mM NaCl, 1 mM DTT. A aliquot (200 µl) of this sample was resolved on a Superose 6 HR 10/300 gel filtration column (GE Healthcare) in the same buffer. Column fractions are concentrated ten fold by TCA precipitation, and analyzed by SDS-PAGE followed by Western blotting with anti-Unc45b and anti-Hsp90β antibodies. The Superose 6 column was calibrated using purified proteins: reticulocyte lysate Hsp90, Unc45b^Flag^, and a complex of Unc45b and Hsp90 formed by combining equimolar amounts of the two proteins.

An anti-Unc45b polyclonal rabbit antibody was used for immunoprecipitation of Unc45b from the 30–60% saturation ammonium sulfate fraction of the C2C12 cytosol. One aliquot of the lysate was incubated with 50 µg of the IgG fraction of the anti-Unc45b antibody overnight at 4°C, another with buffer alone. Protein A agarose beads (50 µl 1∶1 suspension) were added and incubated for 1 hr at 4°C. The Protein A beads were washed with 1 ml of 10 mM Imidazole, 150 mM NaCl, 0.05% NP-40 for 30 min and twice for 10 min at 4°C on a rotating rocker followed by a final wash in the same buffer without detergent. Bound proteins are eluted into SDS-PAGE gel loading buffer and analyzed by SDS-PAGE and Western blotting with anti-Unc45b and anti-Hsp90β antibodies.

### Folding Analysis of Unc45bFlag/Hsp90 complex

Ten microliter translation reactions containing newly synthesized smooth muscle MD::GFP are incubated with 0.2 µg Unc45b^Flag^ or 0.4 µg of Unc45b^Flag^/Hsp90 complex isolated from C2C12 cells for one hour at 25°C. Reactions are divided into two equal aliquots and diluted two fold with SDS-PAGE, or native-PAGE gel loading buffers, and resolved on SDS or native gels, followed by autoradiography.

The native gel electrophoresis is a modified Laemmli Tris-Glycine electrophoresis system that lacks sodium dodecyl sulfate [19]. The stacking gel is 5% acrylamide in 62.5 mM Tris-HCl pH 6.8 buffer and the running gel is 10% acrylamide in 375 mM Tris-HCl pH 8.8. The running buffer is 25 mM Tris-HCl, 192 mM Glycine pH 8.3, and sample-loading buffer is 50 mM Tris-HCl pH 8.0, 10% glycerol and 0.01% bromophenol blue. Sample were diluted at least 5 fold into loading buffer and a maximum of 2 µl of a translation reaction was used per well to avoid overloading. Electrophoresis was for 3 hr at 20–25 mA constant current and 4°C with circulating cold water to prevent heating. Gels were fixed and dried before autoradiography.

### Limited Proteolysis of Unc45bFlag

Unc45b^Flag^ (25 µg) was incubated with trypsin (12.5 ng) in 27.5 µl TBS at 22°C. Aliquots (4 µl) were withdrawn at 0.1, 2, 5, 10, 20 min and diluted five fold into hot SDS gel loading buffer, boiled immediately, and analyzed by SDS-PAGE and Western blotting with anti-Unc45b, and anti-Flag antibodies. For pull-down assay, 100 µg of Unc45b^Flag^ in 100 µl TBS was digested with 50 ng of trypsin for 30 min at 22°C and the digest stopped with 0.1 µM PMSF. The digested Unc45b^Flag^ was diluted with 0.5 ml TBS, and rotated with 25 µl anti-Flag agarose beads overnight at 4°C. An equivalent amount of undigested Unc45b^Flag^ was bound to M2 agarose beads. The beads were washed as already described and incubated with 25 µl aliquots of newly synthesized skeletal muscle MD::GFP for 1 hr at 22°C. The beads were washed and bound proteins eluted into 20 µl SDS gel loading buffer and analyzed by SDS-PAGE and autoradiography.

### Electron microscopy

Rotary shadowing of Unc45b^Flag^ and Hsp90 was done as previously described [20]. The proteins were diluted ∼20 fold to 15 µg/ml into 70% glycerol buffered with 0.2 M ammonium acetate (pH 7.3) and sprayed on freshly cleaved mica before transfer to the rotary stage of an Edward evaporator. After evaporation of the buffer, platinum was evaporated from a tungsten filament at a 9° angle onto the rotating mica surfaces. A coating of ∼1 nm of platinum was deposited. A carbon support layer was added and the replica were transferred to 300 mesh copper grids and imaged with a Philips CM12 transmission electron microscope at 60–80 KV. Micrographs were scanned at a sampling of 0.4 nm/pixel with a Nikon film scanner and boxed images of single molecules process with the single particle analysis programs in the EMAN software package [21]. The image processing involved: high and low pass filtration, centering, masking and reference-free classification. Class averages were evaluated and a subset of classes were used as reference images to align and average particles from data sets contained between 300–800 individual particles.

### Proteins

Myosin and myosin subfragments were prepared from adult White leghorn chicken pectoralis muscle as previously described [20]. Polyclonal antibodies reacting with Unc45b were prepared by immunization of New Zealand White rabbits by Panigen (Blanchardville, WI) using their standard protocol.

## Results

### Unc45b is a cytosolic protein that interacts strongly with Hsp90

To investigate the cellular interactions of the putative myosin chaperone protein, Unc45b, the cDNA for striated muscle specific Unc45b was cloned from myotubes of a mouse myogenic cell line (C2C12). A triple-Flag tag sequence was cloned in frame to the 3′ end of the full-length cDNA and inserted into an AdEasy shuttle vector for production of recombinant adenovirus. Adenoviral vectors have proven very effective for expression of recombinant proteins in the C2C12 cell line [15]. The vectors used for the Unc45b^Flag^ expression contain an IRES sequence that directs the expression of GFP downstream of Unc45b^Flag^ message. Confluent C2C12 myoblasts were infected with the replication-defective adenoviral vector and high infection rates were achieved based on GFP fluorescence. The C2C12 myoblasts fused and formed well-differentiated myotubes after infection (data not shown). Unc45b^Flag^ expression in cultured muscle cells does not disrupt differentiation or the assembly of the muscle specific cytoskeleton and therefore, the adenovirus infected cells could be maintained for 4–5 days to maximize the expression and recovery of the recombinant protein.

**Figure 1:**
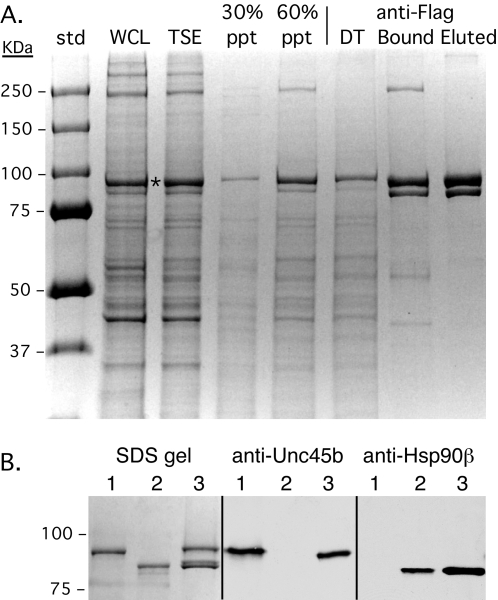
Flag-tagged Unc45b was expressed in C2C12 muscle cells using recombinant adenovirus vectors then extracted, fractionated and affinity purified. A. Adenovirus driven over-expression of Unc45b^Flag^ in C2C12 myotubes results in the accumulation of an abundant 95 kDa band (asterisk) corresponding to Unc45b^Flag^ in whole cell lysates (WCL). The protein is completely extracted into the triton soluble cytosolic fraction (TSE), and fractionates by ammonium sulfate into the 30–60% saturation precipitate (60% ppt). Little is found in the 0–30% precipitate (30% ppt). Affinity purification on anti-Flag beads (mAb M2) followed by dissociation with SDS-gel loading buffer (bound) or elution with 3× Flag peptide (eluted) reveals that Unc45b^Flag^ associates with a 90 kDa protein. The lane labeled DT is the drop-through fraction from the anti-Flag beads containing the protein that did not bind. B. Western blots identify the 90 kDa protein as Hsp90. Samples are: Lane 1, purified Unc45b^Flag^; Lane2, pure Hsp90; and Lane 3, Unc45b fraction eluted from anti-Flag beads. The blots were developed with anti-Unc45b and anti-Hsp90β antibodies. The lower protein loads used for the Western blot experiment resolve the Hsp90 into a doublet, demonstrating that both Hsp90 isoforms (α & β) are bound by Unc45b^Flag^. This was confirmed by Western blots with anti-Hsp90α (data not shown). The Unc45b^Flag^ sample in Lane 2 was expressed in bacteria and purified to homogeneity (see Fig. S1).

Myotubes were harvested and the Unc45b was extracted, fractionated and affinity-purified from the cell extracts using the Flag epitope tag (Fig. 1). The protein is found predominantly in the cytosolic fraction produced by Triton extraction and is not associated with the triton insoluble cytoskeleton (data not shown). Unc45b^Flag^ has an actual molecular mass of 107 kDa, but western blotting with anti-Flag antibody shows that it migrates with an apparent molecular weight of ∼95 kDa in SDS PAGE. The prominent band at ∼95 kDa in Figure 1a is the over-expressed Unc45b^Flag^ in the muscle extracts.

Unc45b^Flag^ partitions in the 30–60% saturation ammonium sulfate precipitate, and re-dissolves when dialyzed against isotonic buffers. The protein is affinity purified from this fraction by binding to anti-Flag mAb (M2) beads and recovered by elution with Flag peptide. It consistently isolates as a complex with a smaller ∼90 kDa protein. Unc45 has been shown to contain three N-terminal TPR motifs that are involved in binding Hsp90 [13]. Western blotting with anti-Hsp90β (Fig. 1B) and anti-Hsp90α (not shown) identify the Unc45b^Flag^ binding partner as Hsp90. These results indicate that Unc45b^Flag^ over-expressed in muscle cells is a cytosolic protein that co-purifies through multiple steps as a complex with its binding partner Hsp90. When Unc45b^Flag^ is over-expressed using the adenovirus vector in Cos7 and HEK 293 cells, it also is isolated as a soluble cytosolic complex with Hsp90 indicating that it forms a stable complex with Hsp90 in the cytosol of muscle and non-muscle cells (data not shown).

Unc45b^Flag^ is also readily expressed as a soluble protein in bacteria. We cloned the Unc45b^Flag^ cDNA from the adenoviral expression vector into a pET vector for expression in *E. coli* [22]. Unc45b^Flag^ is efficiently expressed on induction and surprisingly soluble on lysis of the bacteria (Fig. S1). The bacterial expressed Unc45b^Flag^ was purified to homogeneity by ion-exchange chromatography. It does not co-purify with the bacterial Hsp90 homologue, HtpG. The bacterial Hsp90 homologue lacks the C-terminal acidic sequence recognized by the TPR binding motif of eukaryotic Hsp90 co-chaperones [9], [23].

**Figure 2:**
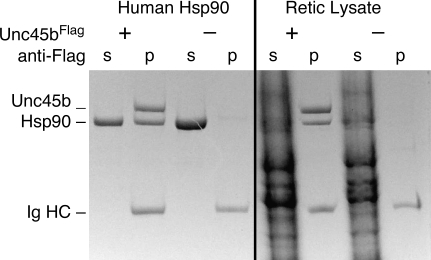
Purified Unc45b^Flag^ binds human and rabbit Hsp90. Unc45b^Flag^ was bound to anti-Flag beads. Aliquots of anti-Flag beads alone or with Unc45b^Flag^ were incubated with purified human Hsp90 or rabbit reticulocyte lysate, and proteins pulled down (p) with the beads and the supernatants (s) were analyzed by SDS-PAGE. Unc45b^Flag^ readily binds purified human Hsp90 in this assay. Unc45b^Flag^ selectively bind Hsp90 when incubated with rabbit reticulocyte lysate (Retic Lysate). The complex recovered from the lysate did not include other Hsp90 binding partners suggesting that the interaction is characterized by a high degree of selectivity.

The purified bacteria expressed Unc45b^Flag^ readily rebinds pure Hsp90 in a pull-down assay (Fig. 2). Complex formation between purified Unc45b^Flag^ and purified Hsp90 suggests that the interaction between these proteins is independent of other factors. Unc45b^Flag^ selectively binds only Hsp90 when incubated with a rabbit reticulocyte lysate. These results indicate that the purified Unc45b^Flag^ retains high affinity and selectivity for Hsp90 in this complex cytosolic fraction. Hsp90 is an ATPase, but the binding interaction with Unc45b is not affected by either ATP or ADP (data not shown).

The over-expression of Unc45b^Flag^ in C2C12 cells indicates it forms a stable cytosolic interaction with Hsp90. Similarly, the purified bacteria expressed protein selectively rebinds Hsp90. Is the endogenous Unc45b in the myotube cytosol also found in a stable complex with Hsp90? To determine the interactions of the endogenous Unc45b in muscle cells, we analyzed cytosolic lysates of cultured C2C12 myotubes by gel filtration and immunoprecipitation. A polyclonal anti-Unc45b antibody was prepared by immunization of rabbits with the purified bacteria expressed protein and shown to detect a single protein in C2C12 myotube extracts (Fig. S2).

**Figure 3:**
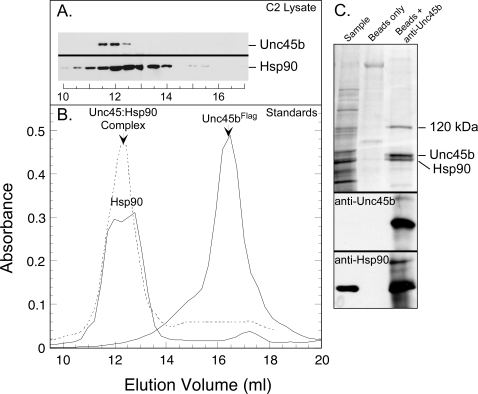
Analysis of Unc45b in muscle cell lysates by gel filration and immunoprecipitation. A cytosolic lysate was prepared from C2C12 myotubes and concentrated by ammonium sulfate precipitation. A. Analysis of the lysate by gel filtration followed by Western blotting shows that the elution of Unc45b and Hsp90 overlap. The muscle Unc45b elutes at an apparent molecular weight that is greater than the 180 kDa Hsp90 dimer. B. The column was calibrated with standards including: Hsp90, Unc45b^Flag^ and a complex of Unc45b^Flag^/Hsp90. Pure Unc45b^Flag^ elutes at a position consistent with a monomeric mass of about 100 kDa. Pure Hsp90 elutes as a broad double peak consistent with its higher mass and conformational heterogeneity. A synthetic complex of Unc45b^Flag^ and Hsp90 elutes as a single peak at the position of the Hsp90, suggesting that the complex is more compact and with less conformational heterogeneity than pure Hsp90. C. Immunoprecipitation of the endogenous Unc45b from the lysate with anti-Unc45b IgG and Protein-A beads shows that Hsp90 is bound to Unc45b in the lysate. This is confirmed by the Western blots with anti-Unc45b and anti-Hsp90. In addition to Hsp90, a 120 kDa band is detected in the antibody pull-down. So, although purified Unc45-Flag is a monomer in solution, the Unc45b in muscle lysates isolates as a cytosolic complex with Hsp90 and potentially one other protein.

Purified Unc45b^Flag^, rabbit Hsp90 and a synthetic complex of Unc45b^Flag^/Hsp90 were individually analyzed by gel filtration to calibrate the column for analysis of the C2C12 lysates (Fig. 3B). Unc45b^Flag^ elutes in a single symmetric peak with an elution volume consistent with a monomer of ∼100 kDa apparent molecular weight. In contrast, Hsp90 elutes earlier from the column in a broad double peak consistent with is larger dimeric mass (∼180 kDa) and heterogenous conformation [23]. The synthetic Unc45b^Flag^/Hsp90 complex elutes in the same position but as a more symmetric peak than the heterogeneous Hsp90 dimer. Analysis of the C2C12 lysate by gel filtration and detection of the endogenous Unc45b and Hsp90 by western blotting of column fractions reveals that the cellular Unc45b does not behave as a monomer. Rather, it elutes at a higher apparent molecular weight and overlaps with the elution position of Hsp90 in the cytosolic extract (Fig 3a). The endogenous Unc45b elutes even earlier from the column than the Unc45b^Flag^/Hsp90 complex used to standardize the column. This is indicative of an interaction between Unc45b and other cytosolic components, including Hsp90.

Immunoprecipitation of the endogenous Unc45b from the C2C12 lysate with the anti-Unc45b antibody demonstrates that it is indeed a complex with Hsp90 and at least one other ∼120 kDa protein (Fig. 3c). These results establish that the endogenous Unc45b in the muscle cytosol exists as a soluble complex with Hsp90 and perhaps one other unidentified protein.

### Unc45b/Hsp90 complex targets the myosin motor domain

Analysis of the myosin subfragments synthesized in rabbit reticulocyte lysate assay have shown that folding of myosin motor domain is the rate limiting step and muscle specific folding factors may be required to complete this step [16]. If Unc45b is indeed a striated myosin specific chaperone, it might be expected to target the myosin motor domain. To test this hypothesis, we synthesized full-length myosin heavy chain, or its subfragments in a coupled transcription translation assay [16]. Fragments retaining light chain binding domain were co-translated with myosin essential and regulatory light chains (cf. Fig. S3). Newly synthesized proteins were incubated with either Unc45b^Flag^/Hsp90 complex isolated from C2C12 cells, or bacterial expressed and purified Unc45b^Flag^. Anti-Flag antibody bound to agarose beads was used to recover the interacting proteins.

**Figure 4:**
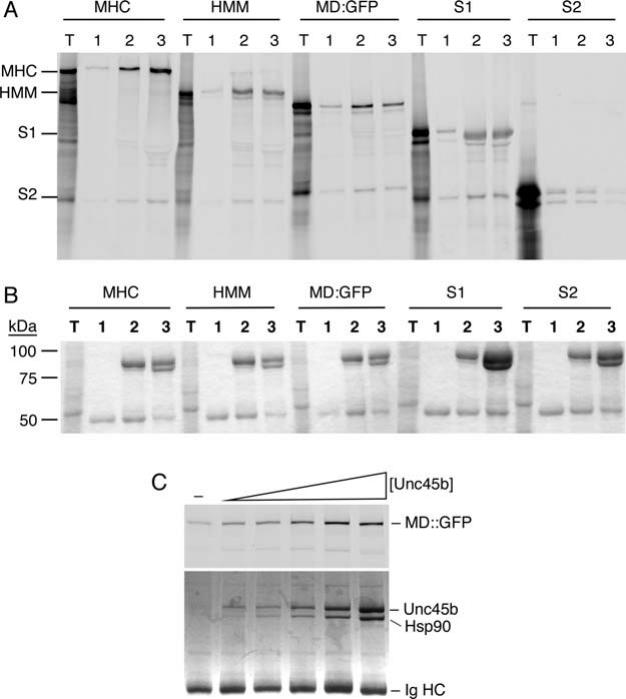
Binding of Unc45b^Flag^ to myosin fragments. To analyze myosin binding activity of Unc45b we synthesized full-length myosin or its subfragments in a coupled reticulocyte lysate synthesis assay and measured pull-down of the radioactive proteins by Unc45b^Flag^ bound to anti-Flag beads. The target proteins included: full length striated muscle myosin (MHC), heavy meromyosin (HMM), a MD::GFP chimera, myosin subfragment 1 (S1) and the subfragment 2 (S2) region of the myosin rod (see also Fig. S3). The newly synthesized radioactive proteins were incubated at 0°C with proteins bound to the anti-Flag beads and analyzed by SDS-PAGE and autoradiography. A. Autoradiography translation mix (T), and the proteins bound to anti-Flag beads alone (1), beads with Unc45b^Flag^ (2) and Unc45b/Hsp90 complex (3). Unc45b^Flag^ and Unc45b/Hsp90 complex bind all myosin subfragments that include the motor domain but not to the S2 region of the rod or the myosin light chains (not shown). B. The stained gel corresponding to the autoradiograph shows that the purified Unc45b^Flag^ binds Hsp90 when added to the lysate. C. The stoichiometry of the Unc45b^Flag^ binding interaction was investigated by titration of the binding of the MD::GFP chimera with increasing concentrations of Unc45b^Flag^. To detect the low concentration of Unc45b^Flag^ recovered in this assay the gel was silver stained before autoradiography. The upper panel shows the autoradiograph detecting the MD::GFP in the pull-down. The lower panel shows the proteins bound to the anti-Flag beads. The amount of Hsp90 detected increases linearly with added Unc45b; whereas, the MD::GFP binding saturates at the higher Unc45b^Flag^ concentration tested. This is consistent with binding of the motor domain by the Unc45b^Flag^/Hsp90 complex. These data are plotted in Fig. S4.

All myosin subfragments containing the myosin motor domain co-precipitate with Unc45b^Flag^, indicating that Unc45b targets the motor domain (Fig. 4a). Unc45b^Flag^ binds the Sk795::GFP chimera (MD::GFP) that encodes a complete, functioning motor domain and lacks any portion of the myosin rod, myosin light chains, and the light chain binding domain [14]. Furthermore, the S2 region of the myosin rod is not bound by Unc45b, and myosin light chains alone do not bind (data not shown).

The stained gels shows that the immunopellets contain Hsp90 in addition to the radioactive myosin subfragments, even when purified Unc45b^Flag^ was added (Fig. 4b). Thus, Unc45b specifically binds the non-native myosin motor domain, and forms a complex containing Hsp90 and motor domain. This is perhaps a trimeric complex of Unc45b, Hsp90, and motor domain. However, excess Unc45b was used in these assays to maximize binding of the myosin subfragments. To look at the apparent stoichiometry of the interaction we titrated Unc45b^Flag^ into the assay and measured Hsp90 and MD::GFP binding (Fig. 4C and Fig. S4). The amount of Hsp90 that is pulled-down with Unc45b increases linearly with the amount of Unc45b added. However, motor domain pull-down fits a hyperbolic binding curve. This suggests that the Unc45b/Hsp90 complex is indeed binding the MD::GFP. Formation of this complex is independent of Hsp90 ATPase activity because Unc45b binds Hsp90 and MD::GFP in the presence of the Hsp90 ATPase inhibitor geldanamycin (data not shown). Furthermore, we have been unsuccessful in developing a pull-down assay for the binding of the motor domain by Hsp90 in the absence of Unc45b, suggesting that Hsp90 alone binds motor domain weakly.

### Unc45b does not bind the native conformation of the myosin motor domain

**Figure 5:**
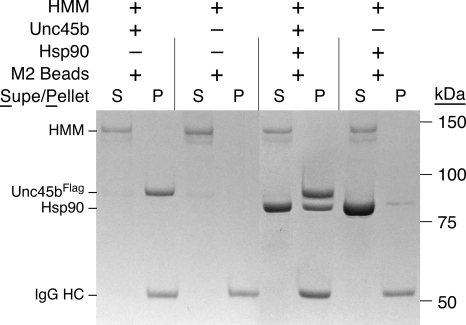
Unc45b does not bind the native myosin motor domain. Native HMM prepared by chymotryptic digestion of myosin was incubated with anti-Flag beads alone and in combination with Unc45b^Flag^ and Hsp90 at 25°C. Native HMM does not bind Unc45b or Unc45b/Hsp90 complex. This is consistent with a chaperone activity for Unc45b^Flag^.

The native HMM subfragment of skeletal muscle myosin that has actin-activated ATPase activity and supports sliding movement of actin filaments is not bound by Unc45b^Flag^ alone or the Unc45b^Flag^/Hsp90 complex (Fig. 5). To assess binding to the native conformation, conditions that readily denature myosin, e.g. dilute protein concentrations at elevated temperatures, were avoided. Under these native conditions Hsp90 is readily bound by Unc45b, but there is no detectable binding of the HMM subfragment to Unc45b^Flag^/Hsp90. Thus, binding of the myosin motor domain by Unc45b^Flag^/Hsp90 complex is limited to non-native conformations of the motor domain, characteristic of a chaperone activity.

### Unc45b/Hsp90 complex enhances the folding of the myosin motor domain

Binding of Unc45b to non-native myosin motor domain is one measure of chaperone activity. However, a better measure is the demonstration of direct participation in motor domain folding. We have demonstrated the utility of the rabbit reticulocyte lysate for analysis of the coupled synthesis and folding of the striated myosin motor domain [14], [16]. The system provides a means to investigate the effect of added factors on folding.

**Figure 6:**
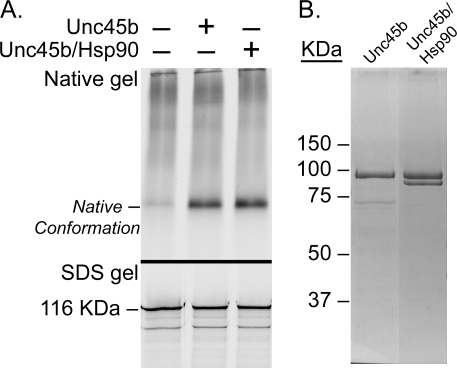
A smooth muscle myosin MD::GFP chimera was synthesized in reticulocyte lysate in the absence and presence of Unc45b^Flag^. Aliquots of translation reaction were analyzed by a native gel eletrophoresis and SDS-PAGE followed by autoradiography. A. The native gel resolves the folded motor domain as a discrete band (Native Conformation). The unfolded motor domain migrates more slowly as a diffuse smear is this gel system. The inclusion of Unc45b^Flag^ (bacterial expression) or Unc45b^Flag^/Hsp90 complex (myotube expression) to the synthesis assay dramatically enhances the formation of native conformation. The SDS gel (lower panel) shows that the levels of MD::GFP synthesis and stability are unaffected by the addition of Unc45b. B. SDS PAGE of the purified proteins added to the reticulocyte lysate: Unc45b^Flag^, and the Unc45b^Flag^/Hsp90 complex.

Smooth and non-muscle myosins have been successfully expressed in non-muscle expression systems [17], and might be expected to fold to a considerable extent in the reticulocyte lysate as well. We developed a vector for *in vitro* expression of a smooth muscle MD::GFP chimera (Sm_795_GFP) identical to the striated muscle chimera. The synthesis and folding of this MD::GFP chimera in a reticulocyte lysate was monitored by native gel electrophoresis (Fig. 6).

Only a small fraction (<10%) of the smooth muscle MD::GFP chimera is properly folded in the untreated reticulocyte lysate. The native MD::GFP conformation in this gel system appears as a discrete, faster migrating band [24]. Much of the nascent and unfolded protein is distributed in diffuse, slowly migrating bands. Addition of purified bacteria expressed Unc45b^Flag^ or the myotube expressed Unc45b^Flag^/Hsp90 complex dramatically enhances the extent of MD::GFP folding that is apparent as a shift of radioactivity to the faster migrating band characteristic of the native conformation. This shift is not a consequence of increased synthesis or enhance stability of the smooth muscle MD::GFP protein. The SDS PAGE analysis of the synthesis reactions shows that the level of incorporation is unaffected by the addition of Unc45b^Flag^. This assay demonstrates that Unc45b has chaperone activity involved in the *de novo* folding of the myosin motor domain.

### Structural organization Unc45b

The sequence of Unc45b suggest that it can be divided into at least three homology regions: three TPR motifs on the N-terminus, a C-terminal ∼420 residues UCS domain that has a myosin binding function, and a large central region of uncertain function that includes regions of limited homology to β-catenin (armadillo repeats). Most models of Unc45 portray the protein as comprised of three independent modular domains based on these homology regions (cf. Fig. S3) [4]. To test the model we probed the structure of Unc45b by two means: 1) limited proteolysis of the protein to detect protease resistant domains, and 2) electron microscopy.

**Figure 7:**
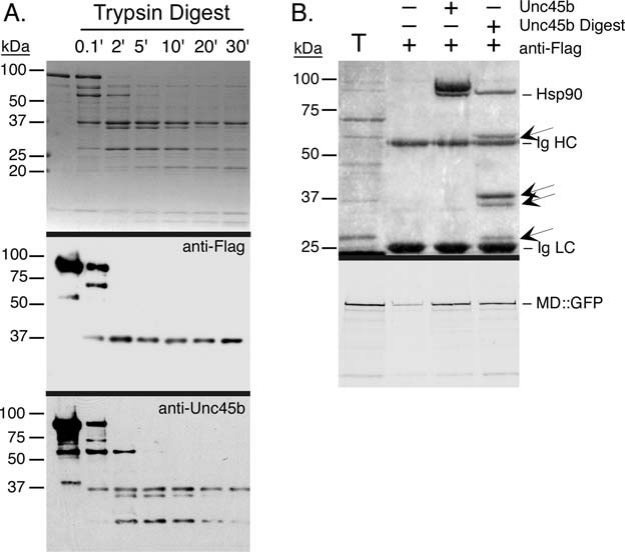
Characterization of the domain structure of Unc45b by limited proteolysis. A. Time course of the digestion of Unc45b^Flag^ with trypsin reveal a ∼37 kDa fragment that appears early in the digest and is relatively stable for up to 30 min. Other initial fragments include a 60 kDa fragment that disappears with the appearance of a 34 kDa and 26 kDa fragment. Western blotting with anti-Flag mAb shows that the 37 kDa fragments retains the C-terminal Flag epitope and corresponds most of the UCS domain. The anti-Unc45b antibody identifies the four early fragments and monitors their disappearance. B. Pull-down assay with anti-Flag beads to determine if the 37 kDa fragment bearing the Flag tag is still capable of binding the myosin motor domain synthesized in the reticulocyte lysate. The fragments produced by 2 min trypsin digestion were incubated with skeletal muscle MD::GFP chimera. The upper panel is the stained gel and the lower panel the autoradiograph. The 60, 37, 34 and 26 kDa Unc45b^Flag^ fragments (marked by arrows) are all pulled-down along with the 37 kDa fragment by the anti-Flag beads. Not only do they all remain associated with the 37 kDa fragment, the protease clipped protein retains myosin motor domain and Hsp90 binding activity comparable to intact Unc45b^Flag^. Lane T is the translation mix alone.

Limited proteolysis with trypsin produced a pattern of fragments that included a 37 kDa fragment that is stable for up to 30′ min of digestion (Fig. 7a). More transient fragments of 60, 34 and 26 kDa are produced in addition to the relatively stable 37 kDa fragment. Western blotting with the anti-Flag mAb reveals that the 37 kDa fragment contains the C-terminal Flag epitope, indicating that it corresponds to much of the UCS homology sequence. The 60 kDa fragment is present only for the first 2 min of the digestion and the appearance of the 34 and 26 Kda fragments and other smaller fragments coincides with the disappearance of the 60 kDa fragment. Chymotrypsin also produces a relatively stable 37 kDa fragment that retains the Flag epitope and another stable 50 kDa fragment (data not shown). These data indicate that the protein has a compact protease resistant conformation particularly in the region near the C-terminus corresponding to the UCS myosin-binding domain.

To evaluate if the C-terminal 37 kDa fragment bearing the Flag tag is a compact independent domain that retains motor domain binding activity, we digested Unc45b^Flag^ with trypsin for 2 min and used the fragments to pull-down newly synthesized skeletal MD::GFP (Fig. 7b). The Unc45b^Flag^ digest was comprised of the 60, 37, 34 and 26 kDa fragments and did not contain any full length Unc45b^Flag^. These four fragments remained associated with the Flag-tagged 37 kDa fragment in the pull-down assay, and they retained the same level of motor domain and Hsp90 binding activity as an equivalent amount of undigested Unc45b^Flag^. Therefore, the Hsp90 binding TPR motifs on the N-terminus remain associated with the C-terminal UCS domain in the trypsin clipped protein. These data are inconsistent with a model of the protein as three independent modular domains. Instead, the results indicate extensive folded interactions that span the full length of the molecule and include surface accessible and protease sensitive loops that do not mark domain boundaries.

**Figure 8:**
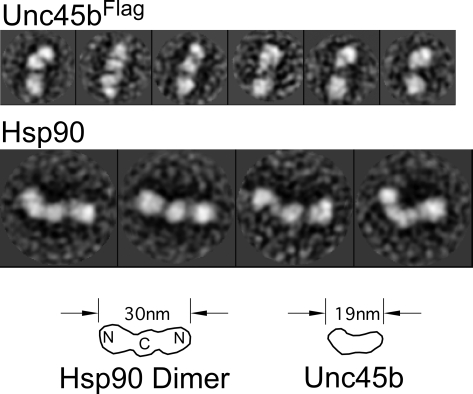
Gallery of Unc45b and Hsp90 molecules contrasted by rotary shadowing with platinum and imaged by electron microscopy. Each image is the class average of 25–50 particles that were selected by reference-free classification of several hundred individual particles and then aligned. These characteristic views of the 105 kDa Unc45b monomer reveal a molecule that is ∼19 nm long and 5 nm wide. Many images show at least two domains, but this interpretation is limited by the resolution of the rotary shadowing contrasting method. Class averages of Hsp90 dimers (∼180 kDa) are shown for comparison. Hsp90 is a 30 nm long dimer with a C-terminal dimerization domain and N-terminal ATPase domains linked by flexible regions.

Finally, the structure of Unc45b^Flag^ was visualized by electron microscopy after rotary shadowing with platinum (Fig. 8). The images of single molecules produced with this contrasting method were analyzed using reference-free classification and averaging techniques to reveal an extended and slightly curved, rod-shaped molecule that is about 19 nm long. There is some suggestion of substructure, but the interpretation is limited by the metal replica method used to contrast the protein. Purified human Hsp90 was analyzed in parallel with Unc45b^Flag^ using the same imaging and single particle averaging techniques. The Hsp90 is a dimer of the ∼90 kDa subunits that associate via a C-terminal dimerization domain. The extended nucleotide free conformation has been published and the images shown here correspond well with the know structure of this protein [25], [26]. We have not been successful at imaging the complex between Unc45b^Flag^ and Hsp90. Nonetheless, the protease digestion experiment and these images provide a working model for Unc45b that suggests an extended molecular structure with extensive inter-domain interactions.

## Discussion

Folding and assembly of striated muscle myosin is a regulated pathway mediated by molecular chaperones [1], [14], [16], [27], [28]. Myosin motor domain folding is assisted in striated muscle cells by the general chaperones Hsp90 and Hsc70 and muscle specific co-factors. We show here that mammalian Unc45b is a cytosolic protein that forms a stable complex with the chaperone Hsp90, selectively binds the unfolded conformation of the myosin motor domain, and promotes *de novo* motor domain folding. These results indicate that Unc45b is a muscle specific co-chaperone that promotes myosin folding.

Genetic interactions suggest a fundamental role for Unc45 in myosin assembly in invertebrates and vertebrates [2], [4], [5]. There is a single invertebrate *unc-45* gene in *C. elegans* and *Drosophila*. Temperature sensitive alleles of this gene disrupt thick filament assembly in the striated muscles at non-permissive temperature [4], [5]. Vertebrates express two distinct paralogs of *C. elegans unc-45* [7]. One, *unc45a,* is expressed generally in all tissues and the other, *unc45b,* is expressed only in striated muscle [8]. Knockdown of Unc45b protein levels results in paralysis and cardiac dysfunction in zebrafish embryos correlated with a loss of myosin filaments in sarcomeres. We have now shown that Unc45b is an active component of the chaperone machinery associated with the folding of the myosin motor domain providing a biochemical explanation of the genetic phenotypes.

The Unc45b interaction with Hsp90 is highly selective. The reticulocyte lysate contains about 3–5 µM Hsp90 that is generally found as a complex with an array of Hsp90 interacting co-chaperones [29]. Unc45b binding to Hsp90 in the reticulocyte lysate is surprisingly free of other Hsp90 interacting proteins suggesting that the interaction is independent of other components of the Hsp90 chaperone machinery present in the lysate. The endogenous Unc45b in muscle cell lysates also interacts with Hsp90 and isolates as a complex with the chaperone. Another ∼120 kDa protein component that has not been identified was also found associated with the Unc45b/Hsp90 complex. Given the selectivity of binding in the retiulocyte lysate, this interaction might be important in muscle. This new component was not detected when Unc45b^Flag^ was over-expressed in muscles cells. The adenovirus induced expression produces up to 100 fold more Unc45b^Flag^ than the levels of endogenous Unc45b found in these cells. Hsp90 is abundant in muscle amounting to 1–3% of the total protein and is present in excess of the Unc45b^Flag^ produced by over-expression. In contrast, the amount of the 120 kDa protein might be limiting and below the detection limit in the over-expressed Unc45b^Flag^/Hsp90 complex we isolated from the muscle cells.

The Unc45b/Hsp90 complex selectively binds the unfolded myosin motor domain but not native myosin subfragments. Therefore, the Unc45b^Flag^/Hsp90 complex has a binding preference consistent with molecular chaperone activity. However, binding of non-native myosin does not necessarily place the Unc45bFlag/Hsp90 complex on the myosin folding pathway. The stimulation of smooth muscle motor domain folding in a *de novo* synthesis assay clearly places this complex on a pathway to myosin maturation. Addition of pure Unc45b^Flag^ or the Unc45b^Flag^/Hsp90 complex dramatically enhances the accumulation of the native conformation by conversion of the unfolded protein to the compact folded conformation.

In a mechanistic characterization of this reaction presented elsewhere, we demonstrate that both vertebrate isoforms of Unc45 behave as activators of the Hsp90 dependent folding of the smooth muscle myosin motor domain [24]. These results place Unc45b and Hsp90 on the myosin maturation pathway. However, in that study we also show that neither isoform supports the folding of the striated muscle motor domain. So, while the Unc45b/Hsp90 complex is sufficient for smooth muscle motor domain folding other components are still needed to complement the folding of the striated muscle myosin motor domain in this assay. These factors are probably involved in regulating myosin folding *in situ*.

The levels of UNC-45 in *C. elegans* appear to be tightly controlled by ubiquitinylation and turnover [30], and it has been suggested that UNC-45 regulates sacomere assembly through myosin ubiquitinylation and degradation [31]. We did not observe enhanced turnover of Unc45b^Flag^ as a consequence of forced over-expression of the protein in the C2C12 myotubes. Furthermore, there was no disruption of the differentiation program or reduction in the assembly of the striated muscle myosin in the C2C12 myotubes promoted by over-expression of Unc45b^Flag^. It is possible that the ubiquitin-linked regulatory pathway was overwhelmed by the level of expression induced here. Alternatively, tight regulation of Unc45b levels might not be an element of the vertebrate regulation system. Nonetheless, we have shown here that a primary activity of Unc45b is as a positive effector of myosin folding.

Finally, we have shown that Unc45b is a monomer in solution with a compact rod-shaped structure by EM. The molecule has been represented as modular based on the division into three homology regions corresponding to the N-terminal TPR motifs, a large central region of uncertain function, and the C-terminal UCS domain [4]. Partial protease digestion produces a discrete C-terminal 37 kDa fragment containing most of the UCS homology region. However, in pull-down assays the flag-tagged 37 kDa fragment remains associated with the other proteolytic fragments, and the fragments retain the same level of motor domain and Hsp90 binding activity as an equivalent amount of undigested Unc45b^Flag^. These results are similar to what is seen with myosin subfragment-1 (S1). Exposed surface loops in the S1 structure are clipped by a variety of proteases producing discrete fragments without disrupting the structure or binding activity of the protein [32]. Therefore, the Hsp90 binding TPR motifs on the N-terminus of the trypsin cleaved Unc45b^Flag^ remain associated with the C-terminal UCS domain, suggesting extensive folded interactions that span the full length of the molecule. These results blur the boundaries between the homology regions and provide a tantalizing first glimpse at this interesting Hsp90 dependent co-chaperone.

## Supporting Information

Figure S1 Expression and purification of bacteria expressed Unc45b^Flag^. The pET21-Unc45a-^Flag^ vector was transformed into *E. coli* BL21 (DE3) Codon Plus and grown aerobically at 37°C then induced with 5 mM IPTG. SDS PAGE analysis shows that Unc45b^Flag^ is highly expressed in lysates after IPTG induction (I) compared to the uninduced (U) bacteria. The protein is in the supernant (S) when cells are lysed under native conditions with very little insoluble Unc45b^Flag^ in the pellet (P). The protein was dialyzed against 150 mM NaCl, 5 mM EDTA, 1 mM DTT and 25 mM Tris-HCl, pH 8.0, applied to a Tricorn Mono-Q 10/100 GL column and eluted with a linear 0.15–1.0 M NaCl gradient. The Unc45b^Flag^ containing fractions were pooled, concentrated, and further purified by gel filtration on a Superose 6 10/300 GL column (GE Healthcare). The protein has hydrodynamic properties consistent with a monomer in solution. Unc4b^Flag^ migrated just below the 100 kDa molecular weight marker of the SDS PAGE system and the final preparation was >98% pure.
(4.78 MB TIF)

Figure S2 Characterization of the anti-Unc45b polyclonal antisera. A. Western blot developed with anti-Unc45b of purified Unc45b^Flag^ protein samples shows the antibody is sensitive to less than 10 ng of antigen. Western blot of lysates of rabbit reticulcyte lysate (RRL), Human HEK 293 cells (293), C2C12 myoblasts (MB) and C2C12 myotubes (MT) shows that the antibody detects a single band in the mouse myotubes lysate. It does not crossreact with the general isoform of Unc45 (Unc45a) found in non-muscle cells or undifferentiated myoblasts. B. The time course of the accumulation of Unc45b in whole cell lysates (WCL) and the triton soluble cytosolic extract (TSE) of C2C12 myotubes after induction of differentiation. Unc45b is a cytosolic proteins that accumulates during differentiation of the muscle cells.
(3.55 MB TIF)

Figure S3 Schematic drawings of myosin subfragments used for analysis of the Unc45b binding assay and the homology regions identified in the Unc45b sequence. A. Myosin II subfragments produced by proteolysis are depicted. Protease cleavage in the rod splits myosin into light meromyosin (LMM) and heavy meromyosin (HMM). Further cleavages releases the S2 subfragment of the rod from the myosin heads (S1) that contain the motor domain (MD) and myosin light chain binding region. Vectors for expression of these different myosin fragments were designed and used to identify the regions that are bound by Unc45b (Fig. 4). B. The sequence of Unc45b can be divided into at least three homology regions that are depicted in the diagram. Three TPR motifs involved in Hsp90 binding are on the N-terminus of the protein between residues 1–110. This is followed by a large central region (residues ∼111–506) of uncertain function that includes a region of limited homology to β-catenein (arm). On the C-terminal there is a ∼420 residue UCS domain that has a myosin binding function. This region has homology to CRO1 from the filamentous fungus *Podospora anserina* [10] and She4p from *S. cervisiae* [11]. Most models of Unc45 portray the protein as comprised of three independent modular domains based on these homology regions.
(0.41 MB TIF)

Figure S4 Quantitation of Unc45b^Flag^ binding to the myosin motor domain and Hsp90 in the reticulocyte lysate. A. The motor domain binding by Unc45b fits a hyperbolic binding curve with apparent K_D_ = 510 nM. B. The amounts of Hsp90 and Unc45b^Flag^ in the pull-down assay each scale linearly with input Unc45b^Flag^ suggesting that they exist as a complex at all concentration tested. These data suggest that the myosin motor domain is bound by a complex of Unc45b^Flag^ and Hsp90.
(3.43 MB DOC)
