# Supplementary figures and images for: Unc45b Forms a Cytosolic Complex with Hsp90 and Targets the Unfolded Myosin Motor Domain
Source: PLoS One. 2008 May 14;3(5):e2137. doi: 10.1371/journal.pone.0002137 (PMC2377097; doi:10.1371/journal.pone.0002137)

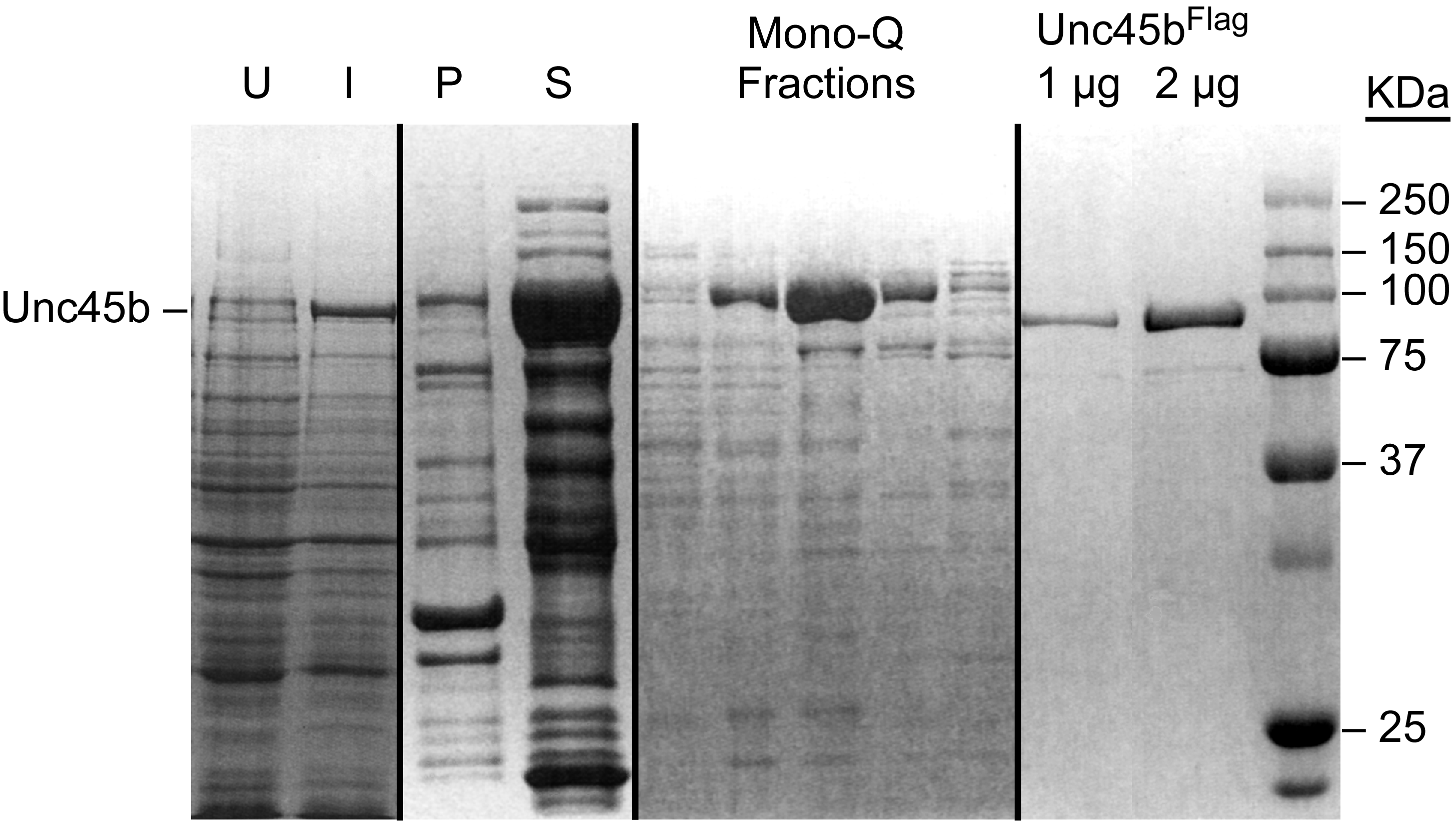

Supplement: Figure S1 — Expression and purification of bacteria expressed Unc45bFlag. The pET21-Unc45a-Flag vector was transformed into E. coli BL21 (DE3) Codon Plus and grown aerobically at 37°C then induced with 5 mM IPTG. SDS PAGE analysis shows that Unc45bFlag is highly expressed in lysates after IPTG induction (I) compared to the uninduced (U) bacteria. The protein is in the supernant (S) when cells are lysed under native conditions with very little insoluble Unc45bFlag in the pellet (P). The protein was dialyzed against 150 mM NaCl, 5 mM EDTA, 1 mM DTT and 25 mM Tris-HCl, pH 8.0, applied to a Tricorn Mono-Q 10/100 GL column and eluted with a linear 0.15–1.0 M NaCl gradient. The Unc45bFlag containing fractions were pooled, concentrated, and further purified by gel filtration on a Superose 6 10/300 GL column (GE Healthcare). The protein has hydrodynamic properties consistent with a monomer in solution. Unc4bFlag migrated just below the 100 kDa molecular weight marker of the SDS PAGE system and the final preparation was >98% pure. (4.78 MB TIF) [file pone.0002137.s001.tif]

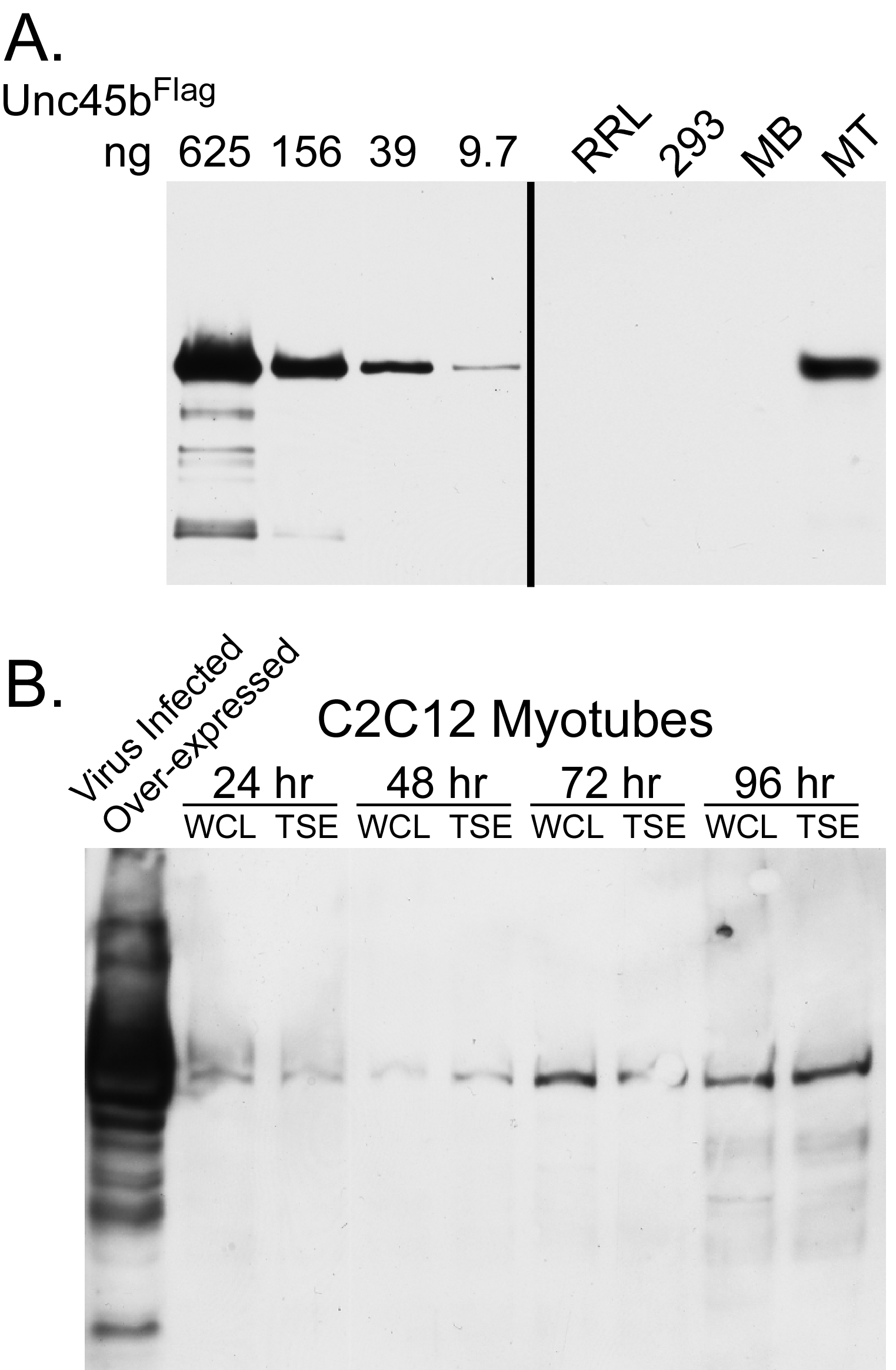

Supplement: Figure S2 — Characterization of the anti-Unc45b polyclonal antisera. A. Western blot developed with anti-Unc45b of purified Unc45bFlag protein samples shows the antibody is sensitive to less than 10 ng of antigen. Western blot of lysates of rabbit reticulcyte lysate (RRL), Human HEK 293 cells (293), C2C12 myoblasts (MB) and C2C12 myotubes (MT) shows that the antibody detects a single band in the mouse myotubes lysate. It does not crossreact with the general isoform of Unc45 (Unc45a) found in non-muscle cells or undifferentiated myoblasts. B. The time course of the accumulation of Unc45b in whole cell lysates (WCL) and the triton soluble cytosolic extract (TSE) of C2C12 myotubes after induction of differentiation. Unc45b is a cytosolic proteins that accumulates during differentiation of the muscle cells. (3.55 MB TIF) [file pone.0002137.s002.tif]

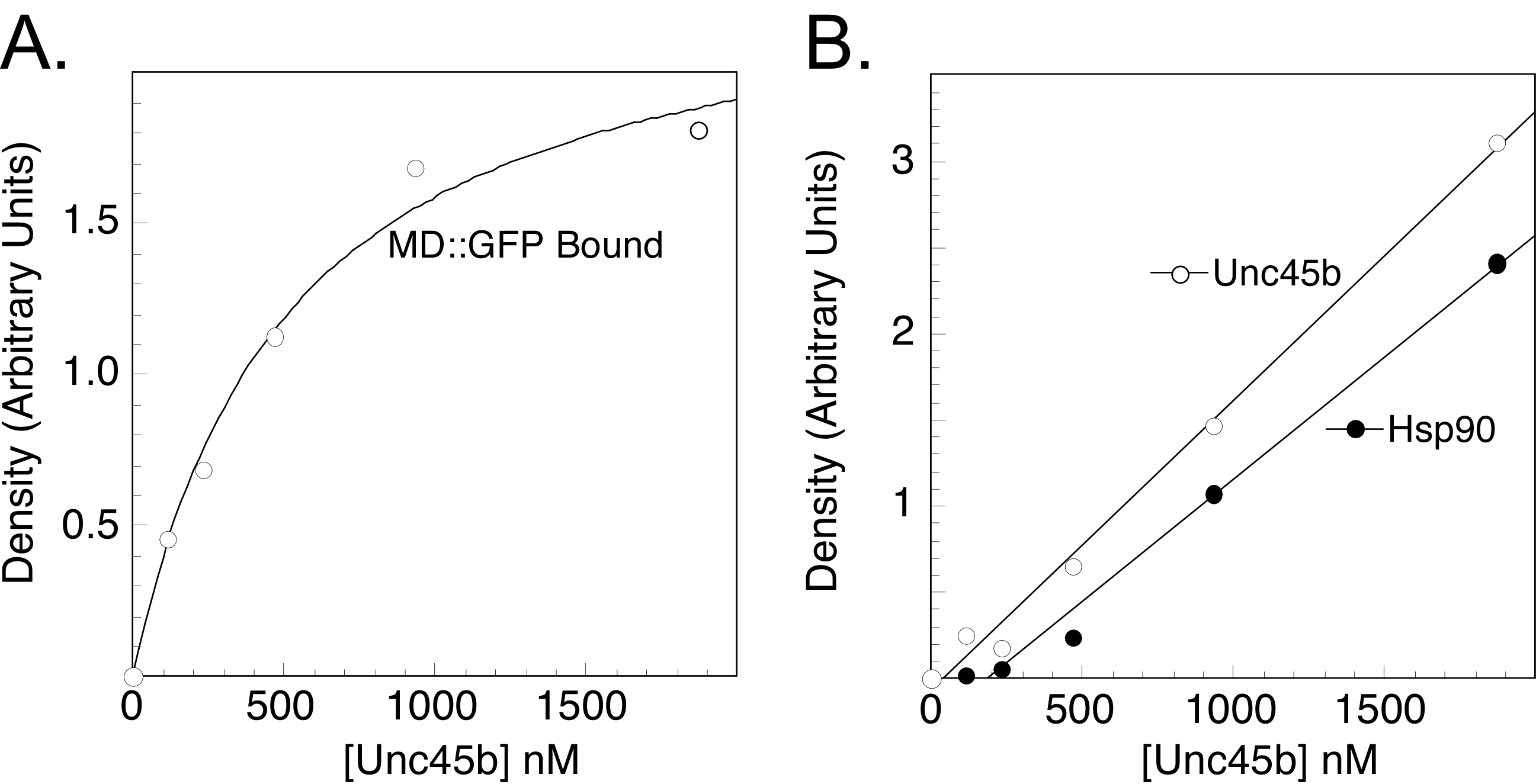

Supplement: Figure S4 — Quantitation of Unc45bFlag binding to the myosin motor domain and Hsp90 in the reticulocyte lysate. A. The motor domain binding by Unc45b fits a hyperbolic binding curve with apparent KD = 510 nM. B. The amounts of Hsp90 and Unc45bFlag in the pull-down assay each scale linearly with input Unc45bFlag suggesting that they exist as a complex at all concentration tested. These data suggest that the myosin motor domain is bound by a complex of Unc45bFlag and Hsp90. (3.43 MB DOC) [file pone.0002137.s004.tif]
